# Presentation of pharmacological content in crime novels between 1890 and 2023

**DOI:** 10.1007/s00210-024-03103-w

**Published:** 2024-04-21

**Authors:** Iven H. Möller, Roland Seifert

**Affiliations:** Institute of Pharmacology, Hannover Medical School, Carl-Neuberg-Str. 1, D-30625 Hannover, Germany

**Keywords:** Drugs, Poisoning, Public Health, Literature, Fiction-reality comparison

## Abstract

As there is lack of research on how drugs are presented in crime literature, we read nearly 25,000 pages of crime literature written between 1890 and 2023 to provide an overview on the pharmacological content in this genre. Correct presentation of pharmacological information decreased over time. Misconceptions about certain substances, especially narcotics and anesthetics appear in many of the analyzed examples. Also, in comparison with crime TV series, books are inferior in providing the reader with additional information and pharmacological plausibility. This especially applies for the newer books which contained less additional information than the older ones. In contrast, some books educate their readers. Newer books show a greater variety of substances also introducing recently developed drugs or new ways of application. On the contrary, older books stick to a small selection of well-known substances during that time, especially metals like arsenic and toxins like strychnine. Gender involvement in poisoning is not realistically presented in the novels. Male victims are overrepresented compared to reality. Also, the etiology is commonly presented incorrectly. Poisoning by accident or for suicidal purposes are rarely presented in the novels, despite their significance in reality. Overall, crime novels educate but also misinform their readers. We discuss the consequences of our findings for the individual reader and public health.

**Supplementary Information:**

The online version contains supplementary material available at 10.1007/s00210-024-03103-w.

## Introduction

In times of global public health crises like the recent COVID19-pandemic, science communication becomes more and more important (Matta 2020). Scientific findings lead to concrete action approaches for governments and society (Wirz et al. 2022). To assure general compliance to such actions in the interest of public health, it is essential that scientific information gets presented in the best comprehensible way. Popular science formats like books have always been a great tool to do so (Sørensen et al. 2012). They both entertain the reader and give him the possibility to develop a better understanding of certain topics. In this way, books are a valuable part in building connections between the scientific and the general world.

Beyond that, science communication also occurs in formats one would not necessarily expect. This includes the most popular book genre: crime novels and thrillers (https://zipdo.co/statistics/book-sales-by-genre/, last accessed on 29.01.2024). They often present a variety of drugs to numb or poison people. The presentation of those substances comes frequently along with technical terms suggesting trustworthy information. By being the most accessible way for most people to get in contact with pharmacological information, the stories have the potential to shape the readers image of certain substances and pharmacological principles in general. This could be both a great chance to casually educate them or a potential danger of supplying them with wrong information.

Despite the great range and potential influence, little research has been performed on the presentation of pharmacological content in literature. The existing work only focusses on either one disease (Vázquez-Espinosa et al. 2020) or one specific author (Gerald 1993a). This includes the most successful female crime author of all time, Agatha Christie (Editors of wikipedia.org 2024). In the book “The Poisonous Pen of Agatha Christie,” Michael Gerald gives a detailed overview on how the former nurse introduced her pharmacological knowledge to her work. As this research has already been performed, we decided to exclude the novels of Christie from our analysis but will link the existing research to our findings in the discussion.

The overall aim of our study was to find out how drugs are presented generally in crime novels, also in comparison to other genres like TV series and the reality.

## Material and methods

Suitable authors of the genre were identified to perform further research. We focused on successful authors with a huge range that at the same time were not yet covered in another analysis. Agatha Christie’s work was already covered in previous studies (Gerald 1993a).

Authors from different times and different western countries were included to identify similarities and differences in terms of cultural aspects and time of origin. Table 1 shows our final author selection including their lifetime, their number of copies sold, their most famous character, and in how many languages their work has been translated to.

**Table 1:** The authors

Author	Lifetime	Nationality	Languages translated to	Sold copies	Most famous character
Sir Arthur Conan Doyle	1859–1930	British	70	Over 60 million	Sherlock Holmes
Georges Simenon	1903–1989	Belgian	>50	Over 500 million	Jules Maigret
Jussi Adler-Olsen	1950–present	Danish	40	Over 27 million	Carl Mørck
Jo Nesbø	1960–present	Norwegian	50	Over 55 million	Harry Hole
Simon Beckett	1960–present	British	29	Over 12 million	David Hunter

Table 1 shows the authors covered in this analysis including additional information (Editors of sherlockian.net 2024; Editors of wikipedia.org 2024; Editors of jussiadlerolsen.com 2024; Editors of jonesbo.com 2024; Editors of simonbeckett.com 2024)

After identifying all crime novels and thrillers written by them, they were read in the German translation. Books for children were not covered. To give the best possible overview, every book of the named authors was read from first to last page. This procedure was adopted to guarantee that every case is part of the analysis, and the shown trends are representative for the author. For the work of Simenon, we had to use another practice as his complete work includes more than 1000 books and short stories. Therefore, the fan page “https://www.maigret.de” was used to identify which stories of Simenon have pharmacological content and are, therefore, suitable for our analysis.

### Statistical analysis

**Fig. 1:**
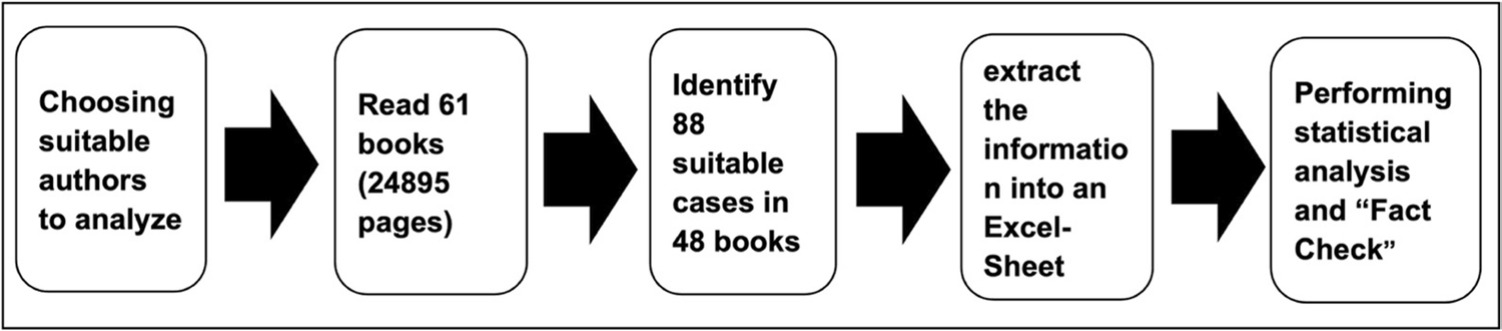
Shows the procedure used for analyzing the novels in a flow chart

In total, 24,895 pages were read, and 88 cases for further analysis were found. This means an average of one case every 283 pages. We found 4 cases in the books of Doyle, 14 in the books of Simenon, 27 in the books of Olsen, 34 in the books of Nesbø, and 9 in the books of Beckett. Figure 1 gives an overview of the reading and analyzation process. A list of all books and page number can be found in Table S1. Out of 61 books, just 13 did not contain any usable case. Fifteen books contained exactly 1 case, and 33 books—including 5 anthologies—contained at least 2 cases. A *case* was defined as a scene in which a pharmacological substance is presented and used beyond their medical indication. Therefore, proper usage of substances performed by professionals to treat diseases was not part of our analysis. If a substance appeared more than once in a book, it was still counted as one case as long it was used the same way. It was counted as a new case if there was a relevant change regarding the analyzed parameters.

To reveal historical trends, the books were divided into two time categories. All books from the authors born around 1900 or before (Doyle and Simenon) were counted as “old,” whereas books from the later born authors (Beckett, Nesbø, and Olsen) were counted as “new/modern.” This leads to cases between 1890 and 1960 for the older and between 1990 and 2023 for the newer books. For division in time categories, the year of initial release was used. In some cases, this differs from the release date of the German translation that can be found in the references.

The cases were drawn out and categorized by the given name, the substance category, substance presentation, way of application, outcome, etiology, gender of offender and victim, whether a mechanism of action was provided, and whether additional information was provided. A fact check of the scenes and further comparison between the authors and the genre was performed after statistical analysis.

### Fact check and genre comparison

For the plausibility check, only information was used the author could have been using too while writing the book. The Poulsson – Lehrbuch der Pharmakologie (“Poulsson – Textbook of Pharmacology”) from 1922 was used to cover the level of knowledge for the older books, whereas modern reference works (Seifert 2019a) and research available on “PubMed” have been used to check the newer ones. For the genre comparison, the work on the German TV series “Tatort” by Ellerbeck and Seifert (2022) and Borchert and Seifert (2023) was used. Reality comparison has been performed with data of the ”GIZ Nord” (“Poison Information Center North”) and “Gitfnotruf München” (“Poison ermergency call Munich”).

## Results

**Fig. 2:**
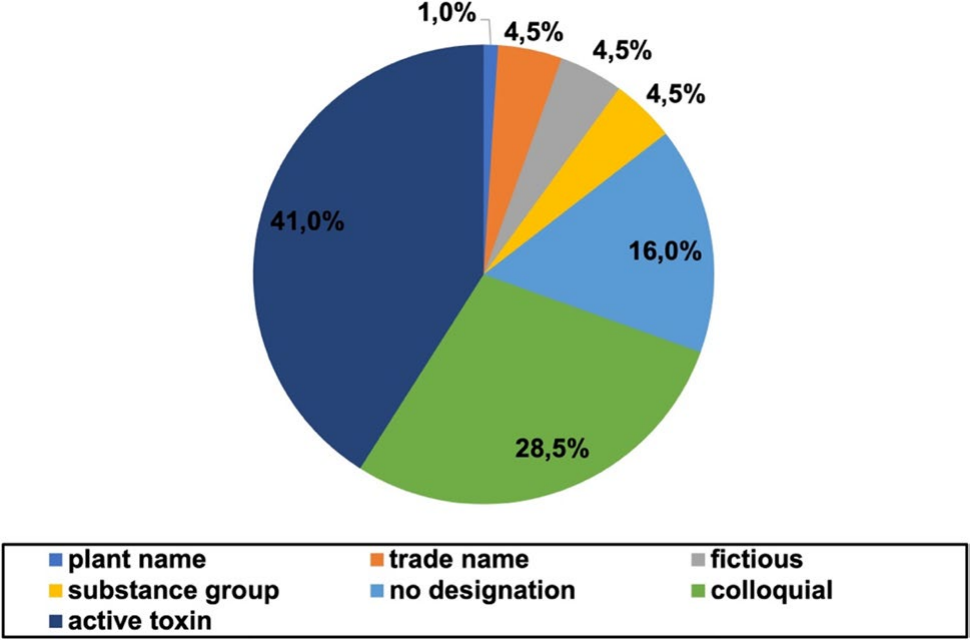
Designation of the active pharmacological substances. The designation of pharmacological substances in all books shown in a pie chart by percentage value

The designation of the pharmacological substances was different between the analyzed novels. Figure 2 shows the different designation of the pharmacological substances in all novels.

Most often, the substances were introduced by naming the active toxin. This was the case for 41% of the findings in total and significantly more in the older novels (Figure S1). The usage of colloquial language such as “heart medication” was found in 28.5% of the cases and appeared more often in the newer novels. This was also the case for the lack of a designation with a total count of 16%. Overall, newer novels presented a greater variety of designation which sometimes impeded with the identification of the particular substance. As examples for designation also the mention of the substance group (4.5%), the usage of fictious names (4.5%), the usage of the trade name (4.5%) and the usage of plant name (1%) were found.

**Fig. 3:**
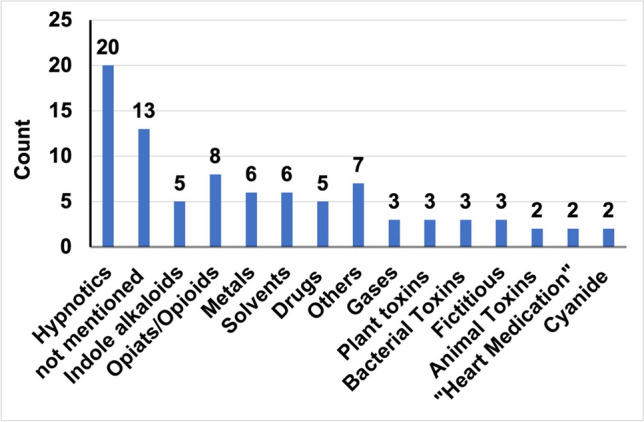
Absolute mentioning of substances by substance categories in all literature, bar chart

All substances we found were categorized as visible in Fig. 3.

Hypnotics, often described as “sleeping pills”, were introduced the most often, with 20 mentions overall. Nineteen of those 20 mentions appeared in the newer novels. The usage of substances in the medical world changes over time; the same appeared for the novels. The increase of hypnotic usage in the fictional world reflects the increase of hypnotic usage in the real world. Since the appearance of benzodiazepines in the 1960s, they rapidly became one of the most frequently prescribed substance groups (Wick 2013). Professionals were pleased to have a presumed safe alternative to the respiratory depressing barbiturates. The discovery of other unwanted effects like addiction some years later led to guidelines to regulate the use. However, they still belong to the most commonly used drugs worldwide (Votaw et al. 2019). The abuse of benzodiazepines became a serious public health problem and is a relevant topic even nowadays (UNDOC 2017). By frequently mentioning the misuse of those substances in their novels, modern crime authors potentially raise the awareness for that.

The second most found substance category was “not mentioned” with 13 entries. Opioids appeared 8 times. The majority of opioid cases (6) ended up with death, showing how dangerous those substances are also in reality. Opioids are involved in 85% of lethal drug overdoses within the European Union (EMCDDA 2019). In America, more than 100 people per day die of opioid related overdose (https://www.cdc.gov/opioids/basics/epidemic.html#:~:text=The%20number%20of%20people%20who ,in%202021%20involved%20an%20opioid., last accessed 01.02.2024). The books again address a serious public health issue here and raise the readers awareness for the danger lies within drug consumption.

Indole alkaloids like strychnine appeared exclusively in the older novels with 5 mentions.

Also, metals were commonly used in the older novels with 5 mentions compared with one in the modern ones. In nearly every case, the metal used was arsenic, and the indole alkaloid of choice was strychnine. As visible in old pharmacology textbooks like the Poulsson (1922a, b, c, d), those two substances were well known also around 1900. The earlier authors seem to stick with substances they already know. In contrast to that, newer novels show a greater variety of substances. Nine different substance categories occur in the older novels, whereas 14 appear in the newer ones. Some agents were used only one single time like thallium in “Takeover” (Olsen 2020) or rodenticides in “Tiere” (“Animals”) (Beckett 2011).

Solvents appeared 5 times in the modern novels and once in the older ones. Drugs were defined as illegal drugs except for opiates/opioids which were covered in an extra category. Drugs appeared exclusively in the newer novels with 5 mentions, same for gases and bacterial toxins with 3 mentions. Other substances—which means substances that only appeared once and were therefore not categorized separately—were mentioned 7 times in total. This appeared for rodenticides, organophosphates, amyl nitrite, potassium chloride, insulin, propofol, and the toxic ingredients of old matchsticks.

Plant toxins were found twice in the new and once in the old novels, same allocation for fictious substances. Animal toxins appeared once for newer and older novels, whereas “heart medication” and cyanide exclusively appeared in the newer novels with two mentions each. An overview of the mention for the substance categories divided into newer and older novels can be found in Figure S2.

The reader also comes in contact with innovations in pharmacology. In the crime novel “Schneemann” (“The Snowman”) by Nesbø (2008), a new kind of pain medication was presented. It was described as a substance from cone snails. The book claimed it as an innovation in the field of pain treatment and as more potent alternative for opioids. Even though the described usage of this substance in the novel was pharmacologically wrong, there was indeed a surge of pain medication research at that time (Safavi-Hemami et al. 2019). It finally turned out that the drugs could not really been used widely due to huge potential of serious unwanted effects (Löschner et al. 2021). However, this example shows that some of the newer books introduce current innovations.

Overall, a huge variety of pharmacological substances gets presented in crime literature, especially in the newer books. Some serious public health issues get addressed here, and the reader can learn something about newly developed drugs. The older books focused on a smaller selection of substances. The reader here has less opportunity to get in contact with new substances and pharmaceutical innovations.

**Fig. 4:**
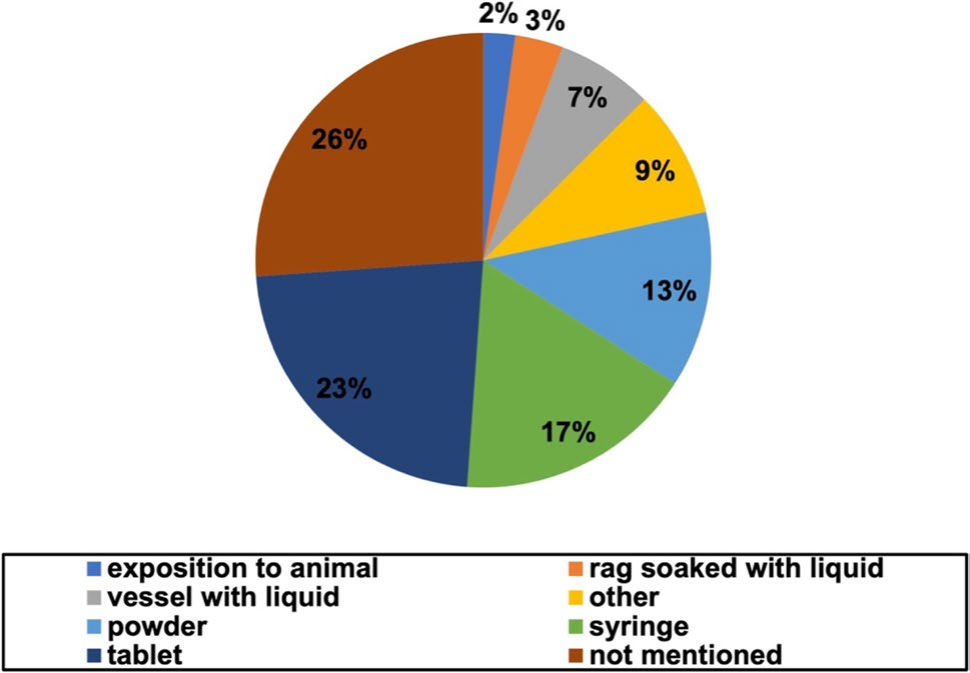
Substance presentation. The pie chart shows the presentation of substances with percentage value for all cases

There were various ways of substance presentation that we found in the novels as visible in Fig. 4.

The leadings category was not mentioned with 26%. No big difference was found between older and newer books (Figure S3). The second most described presentation forms were tablets (“pills”) with 23% and syringes with 17%. Syringes were exclusively mentioned in the newer novels. In 13% of the cases, the application form was powder; in the older novels, they accounted for a share of 50% and were thereby the most common form of application. Additional ways of application found were “other” with 9%, vessel with liquid 7%, rag soaked with liquid 3%, and exposition to animal with 2%. An example for alternative ways of application described as “other” was the description of gases in the surrounding respiratory air (Nesbø 2009).

**Fig. 5:**
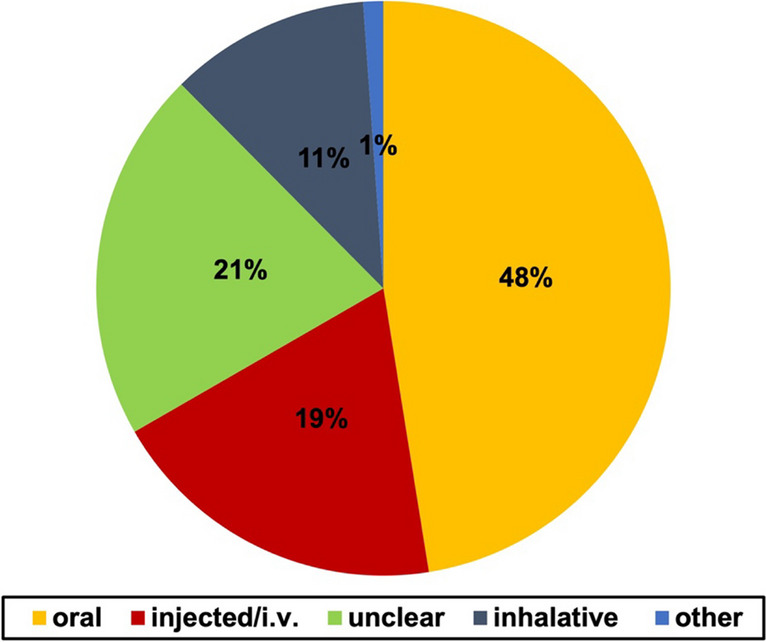
Substance application. Pie chart with percentage value of all cases. The categories are presented with different colors for each application form

Figure 5 shows the application of the substances in all analyzed cases.

The most common way of application was oral with 48%, followed by injected with 19%.

In 21% of the cases, the way of application remained unclear. In 11%, inhalative application was described, and in 1%, other forms were described.

Oral application is not only the most common form in the novels but also in reality (Alqahtani et al. 2021). It has been even more prominent in the older novels. The commonly used substances in older literature naturally came up in the form of powder and here therefore taken orally. However, the oral way is still the most common form also in the newer novels.

The mention of injection has nearly doubled in the newer novels (Figure S4). This reflects true pharmacological progress within the last decades. With the advent of disposable syringes in the second half of the twentieth century (Skaftason et al. 2011) and the scientific progress in the development of biopharmaceuticals (https://www.pfizercentreone.com/insights-resources/articles/what-are-key-trends-and-developments-injectable-drug-formulation-and, last accessed 01.02.2024), this way of application becomes more and more significant in pharmacotherapy.

Again, the newer novels here reflect a real trend in pharmacology which shows that the authors are influenced by scientific developments, at least to a certain degree.

**Fig. 6:**
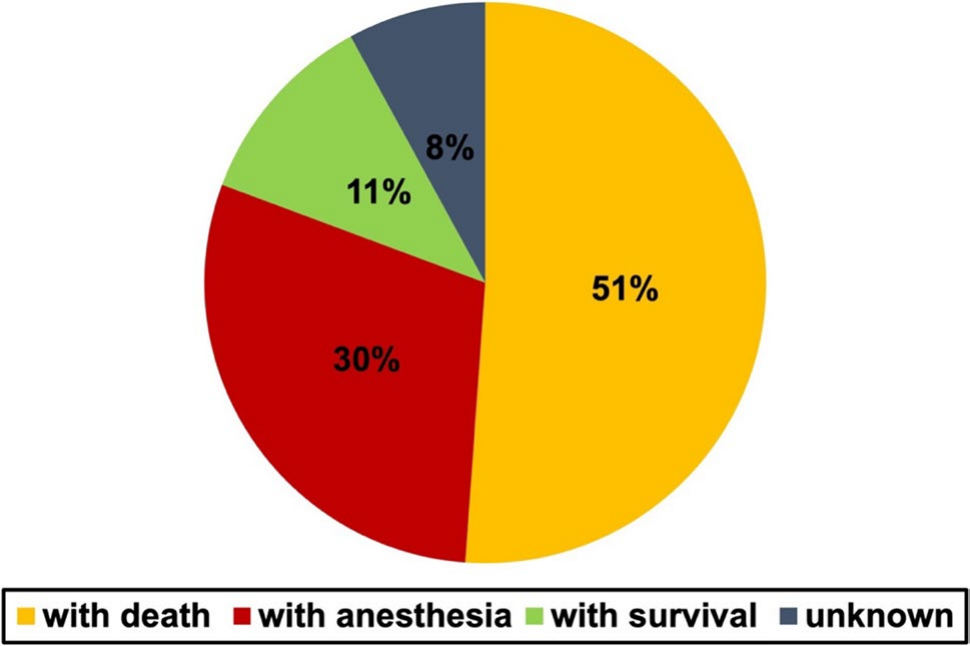
Outcome of poisoning by percentage value in a pie chart

Figure 6 shows the outcome of poisoning by percentage value for all cases (a comparison between new and old cases can be found in Figure S5).

If the target of the person using or delivering the substance was to kill somebody and it succeeded, that was counted as outcome “with death.” If the person survived, it was counted as “with survival.” If a person was intentionally numbed by a substance, this was counted as “with anesthesia,” exceptionally the case the person died. Then this was also counted as outcome “with death.” If the person simply got numbed and survived, it stayed in the category “with anesthesia.” No person was categorized twice. The most common outcome was death with 51%. The survival rate was 11%. Cases of anesthesia were found in 30% of all cases, and in 8% of the cases, the outcome remained unknown.

Regarding the outcome, the books partially reflect trends from reality. The role of stunning people with pharmaceuticals, especially injected ones, has noticeably increased in recent literature. In the newer books, the outcome “with anesthesia” appeared in 36% of the cases compared with only 6% in the older ones. An example for that is “Leopard” (“The Leopard”) by Jo Nesbø (2011). Several people get stunned by an injection of a fictious anesthetic similar to ketamine. Also, in reality, it gets more common to stun people with intravenous drugs. Whereas the usage of oral substances has been a common way to target victims in night clubs, it gets more and more widespread to use injections as an alternative (https://www.theguardian.com/uk-news/2021/oct/19/police-investigate-reports-of-spiking-by-needle-at-nottingham-clubs, last accessed 01.02.2024). Novels might raise the awareness for those new forms of application.

Apart from that, the outcome shown in the novels is also source of misinformation.

With 72% in the newer and 46% in the older novels, most victims of fictional poisoning died. This stands in contrast to reality. According to GIZ Nord (2021), less than 1% of their registered poisoning cases in 2021 ended up with death. The novels in these cases draw a wrong picture of the danger coming from pharmacological substances. In “Eifersucht” (“The Jealousy Man”) (Nesbø 2021) and “Die Larve” (“Phantom”) by Nesbø (2012), an overdose of heart medication led to death. Such descriptions might undermine the trust in safe substances and might lower patience adherence to take them.

**Fig. 7:**
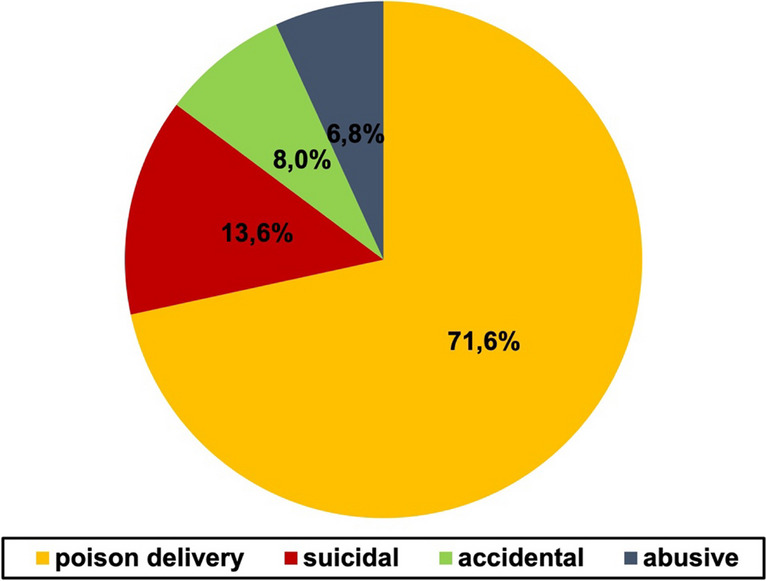
Etiology of poisoning shown in a pie chart. All cases are included. The categories are presented with different colors for each category of etiology that we found. Figure S6 shows the etiology of poisoning for the different time categories analyzed

Figure 7 shows the etiology of poisoning with all analyzed cases included.

In this category, the reader gets misinformed. In 71.6% of the fictional cases, the poison was delivered by another person and not taken by the victims themselves. Suicidal (13.6%), accidental (8%), and abusive use (6.8%) played a minor role. This stands in contrast to reality. *Giftnotruf München* is an emergency number that professionals or laypersons can call in case of acute intoxication. They also monitor the etiology of the cases reaching them (Giftnotruf München 2022a).

Out of 16,000 poisoning cases in adults in 2022, the most common etiology was by accident with about 50%, followed by suicidal with nearly 30%, followed by abuse with about 10% (Giftnotruf München 2022b). Poison delivery was not even listed as an etiology at all. In reality, the danger to get poisoned at home by accident is much higher than getting poisoned by another individual as incorrectly described in the novels. The readers might get more aware regarding external threat but underestimating the much bigger danger in their daily life. In the age group 14–17 years, the most common etiology surveyed by *Giftnotruf München* was suicidal with nearly 50% (Giftnotruf München 2022c). This also is not reflected in the books where suicidal pharmaceutical intake plays a minor role. At least the representation of this has increased. Whereas in the older novels, only 6% of the analyzed cases involved suicide, it was 16% percent in the newer novels. However, the shown etiologies differ a lot from reality and are not helpful in raising public awareness for serious dangers.

**Fig. 8:**
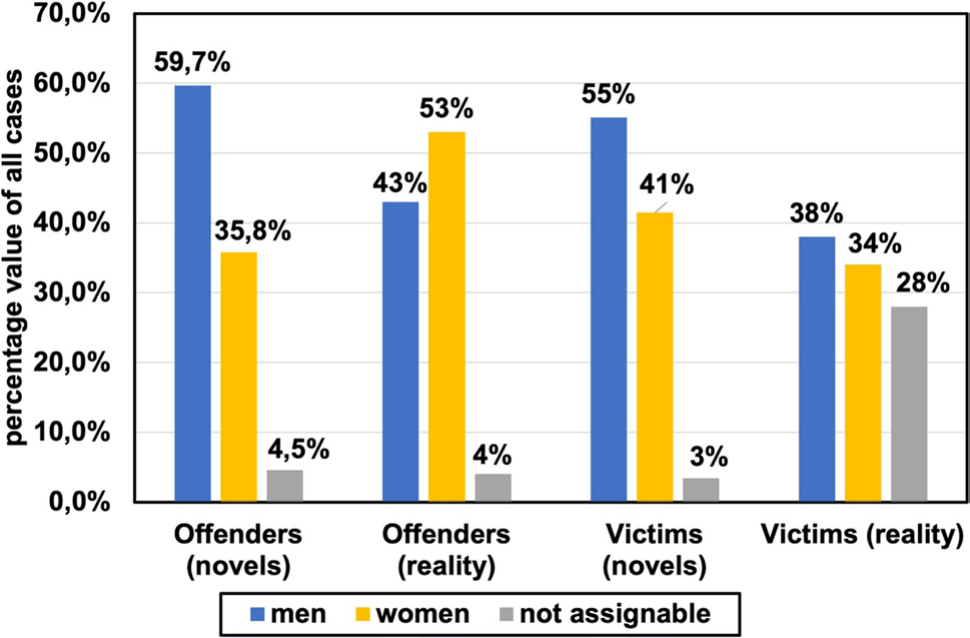
Gender analysis of all cases by percentage value. The genders of offenders and victims involved in criminal poisoning cases are presented. It is shown for the novels we analyzed and data from reality. Males are represented by the blue columns and females by the yellow columns. Not assignable cases are shown with grey columns. Transgender persons were not found in the novels analyzed and are therefore not part of this chart

Figure 8 shows the gender distribution of all analyzed cases. The reality comparison is based on a work of Fuhrmeister (2005). A comparison between older and newer novels can be found in Figures S7 and S8.

Regarding gender in poisoning, the books draw a wrong picture of reality. Over all books, the offenders are mainly male (59.7%), whereas more female offenders are involved in real criminal poising cases (53%) (Fuhrmeister 2005). Also, the victims of poisoning are primarily male in the books (55%), whereas it seems to be nearly even in reality (38% male; 34% female). To a significant extent, the victims’ gender in reality was unclear (28%). In the older books, male had been the victims in 2/3 of all cases. This overrepresentation of men as victims of poisoning may give female readers a wrong security although they are nearly evenly suffering from poisoning cases in reality.

**Fig. 9:**
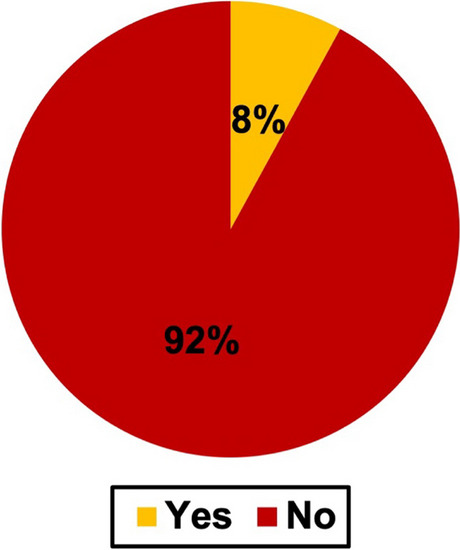
Mechanism of action provided. The pie chart shows if a mechanism if actions was provided. It includes all cases

Figure 9 shows that if a mechanism of action was provided; it includes all analyzed cases.

Among all books, a mechanism of action was not described in 92% of the cases. There was no difference between newer and older novels as shown in Figure S9. Only in 8% of the cases, a mechanism of action was described.

**Fig. 10:**
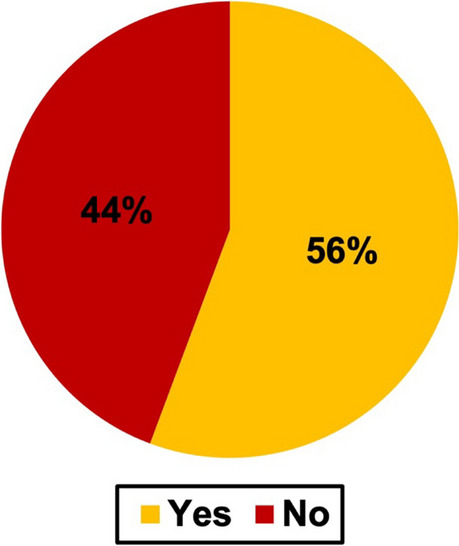
Additional pharmacological information such as on symptoms, latency times or dosage provided. The pie chart shows the percentage value for all cases

Figure 10 shows if the analyzed cases provided additional information or not.

Additional information was defined as pharmacological content such as symptoms, latency times, or dosages. An overview for older and newer novels separately can be found in Figure S10. Overall, additional pharmacological information was found in 56% of the novels which means that 44% of the cases contained no further information.

**Fig. 11:**
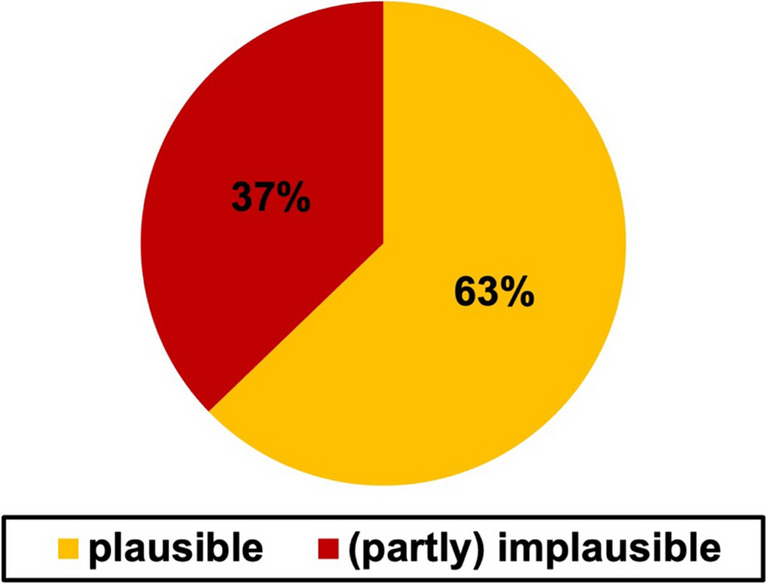
Pharmacological plausibility of all cases by percentage value. The plausibility is shown in a bar chart. A comparison chart between older and newer cases can be found in the supplement (Figure S11)

Figure 11 shows the result of the pharmacological fact check we performed on the fictional cases.

To check the information regarding their pharmacological plausibility, we only used data the authors could have been using to while writing the book. For the older cases, mainly the Poulsson – Handbuch der Pharmakologie (“Poulsson – Textbook of pharmacology”) from 1922 was used (Poulsson, E. 1922a).

For the plausibility check of the newer cases, we used a current pharmacology reference textbook (Seifert 2019a) and current research data from “PubMed.” Overall, 63% of the cases were plausible; 37% contained, at least minor, inaccuracies.

Figure S11 shows that the plausibility decreased over time. In the older books, the rate of correct cases was 72% percent, while it is 60% today. Table 2 shows examples of false presentation. It describes the case and gives a detailed fact check. Table 3 does the same with examples of correct presentation.

**Table 2:** Examples of wrong substance presentation with description of the case and detailed fact check

Year of initial publication, German title (English title), author	Active substance	Content	Fact check
1932, Maigret und der geheimnisvolle Kapitän (Maigret and the Death of a Harbor Master), G. Simenon	Strychnine	A man was poisoned by strychnine in a glass of water. He looks green and dies with twitching limbs. The face only begins to twitch shortly before death (Simenon 1932)	Questionable: Strychnine tastes noticeably bitter even at a dilution of 1:50,000 In addition, acute intoxication leads to the simultaneous contraction of all striated muscles, so that the facial muscles are also affected right from the beginning (Poulsson 1922b)
1892, Das gefleckte Band (The Adventure of the Speckled Band), Sir A.C. Doyle	Snake venom	A man dies from a snakebite within 10 seconds. The poison impresses with its quick effect and probably cannot be traced (Doyle 1892)	Questionable: Snake venom is deadly effective in very low doses, so the lack of detectability is plausible However, the described latency until death is too short (Poulsson 1922c)
2010, Verachtung (Journal 64), J.A. Olsen	Propofol and flumazenil	An injection of propofol quickly renders a person unconscious. Later, the effect is quickly reversed with an injection of flumazenil (Olsen 2012)	Critical: Benzodiazepines bind to the γ-subunit of the GABA-A-receptor, while propofol occupies the β-subunit The effect of benzodiazepines on the GABA receptor can be quickly antagonized by flumazenil, but that of propofol cannot (Seifert 2019b)
2012, Erwartung (The Marco Effect), J.A. Olsen	Unknown narcotic (injected)	Girl gets injection and falls unconscious until awakened. She felt like she was under anesthesia. She felt slightly nauseous afterwards, and it was only after 10 minutes that she was able to walk again (Olsen 2013)	Critical: Suggests easy control and safety of anesthesia without monitoring vital parameters. Real risk of complications, especially if monitoring is lacking, is ignored (Editors of anesthesiologynews.com 2021)
2021, Natrium Chlorid (The Shadow Murders), J.A. Olsen	Sleeping pills (hypnotics)	Dead woman is found in bed with lots of pills around her. There is suspicion of suicide with sleeping pills. No further substances like alcohol involved (Olsen 2021)	Critical: The story takes place in 2020 and suggests that you can commit suicide with an overdose of common sleeping pills. This is not the case (Seifert 2019c)

**Table 3:** Examples of correct substance presentation with additional fact check

Year of initial publication, German title (English title), author	Active substance	Content	Fact Check
2010, Verwesung (The Calling of the Grave), S. Beckett	Cocaine	A man overdoses cocaine only to experience heart attack symptoms. Symptoms: Blood pressure rises, heart palpitations and cardiac arrhythmias. Later dies from the long-term effects of heart failure and pneumonia (Beckett 2012)	Plausible: Cocaine stimulates the release and inhibits the neuronal re-uptake of biogenic monoamines. The increased activation of alpha-1A-adrenoreceptors and beta-1-adrenoreceptors caused by higher monoamine concentrations can trigger the mentioned acute cardiovascular symptoms. The long-term damage mentioned can also be reconciled with cocaine abuse (Richards et al. 2023)
2010, Verachtung (Journal 64), J.A. Olsen	Hyoscyamine/scopolamine/atropine	Murder of several people using henbane extract, substances are named The poison is administered in tea, which victims find bitter tasting A variety of symptoms occur, including constipation, hot flashes, impaired vision and speech, hallucinations, dry mouth, tachycardia and cardiac arrhythmias (Olsen 2012)	Plausible: The alkaloids mentioned are actually contained in henbane, and the bitter taste is also realistic. All symptoms described can be reconciled with intoxication by the substances described (Editors of gizbonn.de 2023)
2013, Koma (Police), Jo Nesbø	Nitrazepam	A man drinks coffee laced with nitrazepam and falls asleep while sitting on guard. After waking up, he feels like he is half asleep for a long time, with a “foggy brain.” Later, the effect is quickly reversed with an injection of flumazenil (Nesbø 2013)	Plausible: Like all benzodiazepines, nitrazepam modulates the GABA-A receptor and has, among other things, a sedative-hypnotic effect. Due to its long half-life of more than 24 hours, typical ADRs such as daytime sedation and confusion can still occur the next day (Seifert 2019b)
1953, Die Eisentreppe (The Iron Stairs), G. Simenon	Arsenic	A man is poisoned with small doses of arsenic through regular meals. He suffers from weight loss, sensation of heat in his throat, stomach pain and heart problems (Simenon 1988)	Plausible: Symptoms consistent with chronic arsenic intoxication (Poulsson 1922d)
1890, Das Zeichen der Vier (The Sign of the Four), Sir A.C. Doyle	“Strychnine-like substance“	A dead person is found. There is a thorn behind his ear. The muscles and face are very tense. The cause is suspected to be a strong, vegetable alkaloid, a strychnine-like substance, which causes tetanus (Doyle 1890)	Plausible: In the context of acute poisoning, fatal tetanic seizures occur (Poulsson 1922b)

**Fig. 12:**
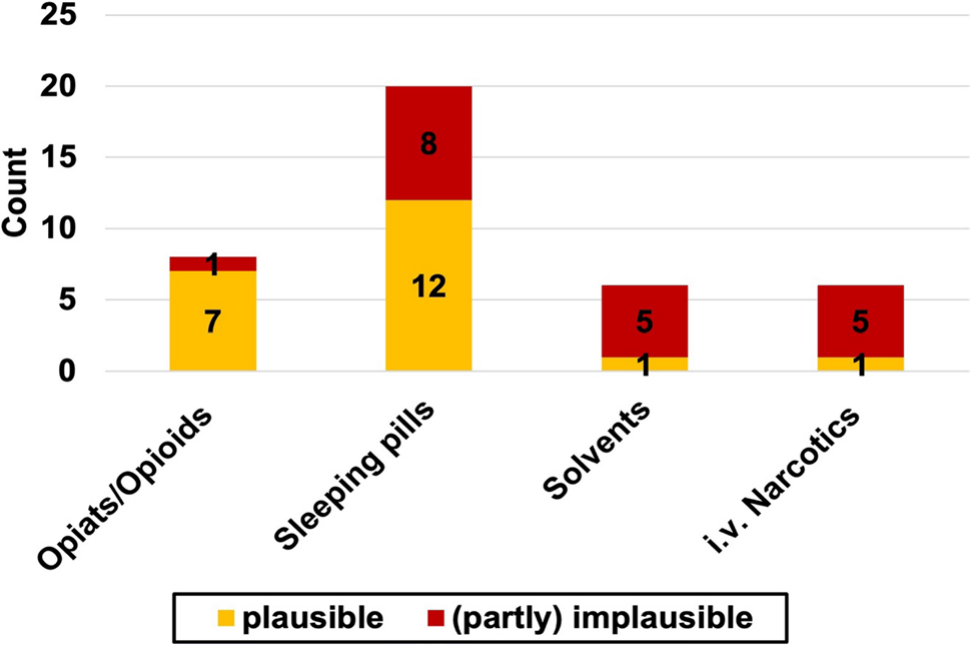
Pharmacological plausibility of commonly used substances. The total count of substances over all novels is shown. The plausible fraction of cases within the column is marked yellow whereas the implausible fraction of cases involving the particular substance is marked red

Figure 12 shows which substances were often source for mistake.

**Fig. 13:**
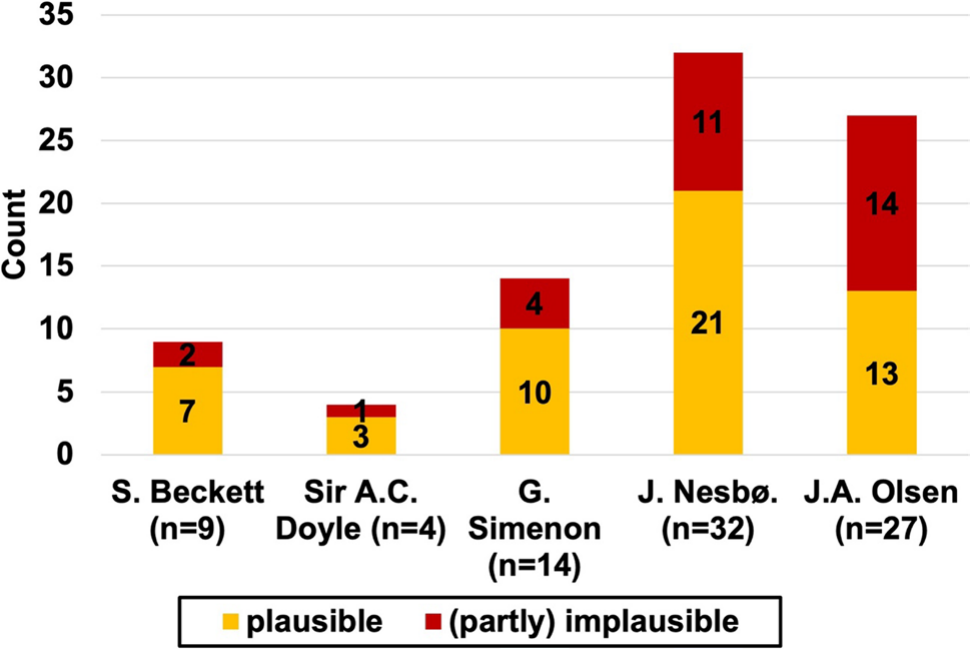
Pharmacological plausibility by author. The chart shows all authors included in the analysis.

Figure 13 gives a comparison between the analyzed authors.

Each column represents all cases of this author we checked the plausibility for. The plausible count of cases is colored in yellow; the implausible in red

## Discussion

### Educating the reader

Literature has always been a great tool to educate the readers about topics. As many people are not in contact with advanced pharmacological knowledge daily, the description of those topics in media like books has a substantial impact on how the audience imagines drugs and poisons to work.

Despite this opportunity to educate the readers and spread pharmacological knowledge in a thrilling setting, the mechanism of action only got explained in a minority of the novels. In most cases the reader gets no further information on what the substances exactly do to the body and how. If a mechanism of action was described like in “Kakerlaken” (“Cockroaches”) (Nesbø 2009), the description was very detailed. The reader gets precise information on how an intoxication with carbon dioxide gradually leads to death. This example shows that it is possible for the authors to implement valuable pharmacological education in their literature and leads to the question why they do not do so on a regular basis.

When it comes to additional pharmacological information this was also missing in the majority of the analyzed cases. In the older novels, it was more likely to get additional pharmacological information (72% vs. 51%). Also, here, the authors miss the opportunity of expanding the readers knowledge on drugs and poisons. It has been quite common in the older books to give at least some extra information like symptoms in “Die Wahrheit über Bebe Dong” (“The Truth About Bebe Dong”) (Simenon 1978) or dosage in “Sonntag” (“Sunday”) (Simenon 1977). In the newer cases, poisoning and drug use are often mentioned in passing. They rarely get described comprehensively like in “Verachtung” (“Journal 64”) (Olsen 2012) where the reader witnesses the whole process of alkaloid intoxication from the first symptoms to death. Overall, newer authors again miss the opportunity to educate the readers about pharmaceuticals especially compared with the older ones. This may lead to a lesser knowledge about substances among the readers nowadays compared to those in the past.

### Reality check

The fact check performed for our analysis showed that the pharmacological correctness in crime literature decreased over time. Although it is easier than ever before to gain valid information by using online research or getting in contact with experts from all over the world with a mouse-click, modern books were lacking correctness. The most named substances in modern crime novels (hypnotics) were also those with a large error ratio. The same applies for solvents and anesthetics.

Common sources for mistakes were false attribution of effect (Olsen 2009/Nesbø 2018), wrong description of symptoms (Simenon 1932), or too short latency (Doyle 1892/Nesbø 2009). Also, wrong antidote use appeared. In “Verachtung” (“Journal 64”) (Olsen 2012), an unconsciousness caused by propofol was rapidly antagonized by Flumazenil. As they interact with different subunits of the GABA-receptor, this is not possible. In fact, there is no proper antidote for propofol (Keymling et al. 2013). This leads to a misconception in the readers mind giving him the idea that there are many potent antidots for common anesthetics like propofol.

In modern books, hypnotics were often used to commit suicide like in “Natriumchlorid” (“The shadow murders”) (Olsen 2021), “Verachtung” (“Journal 64”) (Olsen 2012), or “Der Sohn” “The Son”) (Nesbø 2015). In fact, suicide with old sleeping substances like barbiturates were common and possible as they could lead to a lethal suppression of the respiratory center in the medulla oblongata.

With modern substances like benzodiazepines and non-benzodiazepines (also known as “Z-drugs”), respiratory arrest is hardly possible. The reader gets a wrong picture of those substances here and may therefore reject therapy with those although hypnotics can be helpful, especially in psychiatric emergency situations. Hypnotics are also used together with other substances like alcohol (Beckett 2011 or Nesbø 2010). These combinations are indeed dangerous and could lead to a lethal depression of the respiratory center. However, this danger is not addressed in any of the stories. The reader stays unaware of the danger lies within combining hypnotics and alcohol.

Not further classified drugs to numb people by injection were often used in modern crime literature. They work within seconds (Nesbø 2013), are easy to handle (Beckett 2022), and are used without any monitoring (Olsen 2019). This suggestion of monitor-free anesthesia without any big risks is far apart from reality. Famous persons like Michael Jackson (https://www.health.harvard.edu/blog/propofol-the-drug-that-killed-michael-jackson-201111073772, last accessed 14.02.204) died of unmonitored injections of propofol. The reader should be educated not to experiment with these drugs under wrong preconditions.

However, there were examples of cases where the description was correct and gave the reader the possibility of advancing their pharmacological knowledge. In “Verwesung” (“The calling of the grave”) (Beckett 2012), we found a correct description of cocaine abuse including symptoms of overdosage and possibly lethal long-term effects. Also, the poisonous effects of plant-based alkaloids were described in great detail in “Verachtung” (“Journal 64”) by Olsen (2012). Moreover, examples of correct hypnotic presentation were found. In “Koma” (“Police”) by Jo Nesbø (2013), a stunning case with a benzodiazepine even includes the mention of adverse drug reactions the following day. Those carefully researched examples show that it is possible for an author to give a realistic picture. This way, the reader can learn facts about pharmacology and get a better understanding of a wide range of substances. As also dangers like death and ADRs are shown in the correct examples, it is unlikely that persons would imitate those.

### Author comparison and point of view

Overall, authors from former centuries wrote more plausible books. This surprised us a lot as careful research was way more difficult in the past centuries. The authors had to consult libraries or professionals which is much more time-consuming than using the internet or artificial intelligence as it is possible for today’s content creators. Why mistakes like the misrepresentation of hypnotics then commonly occur in the modern books has to be part of further research. Maybe also professionals have common misconceptions about particular substances. This would have a negative impact on public health and should be investigated in another study.

Although the modern authors did not perform as well as the old ones regarding plausibility, the most correct author by mere numbers was Simon Beckett, a modern author. He often gave detailed information, for example about medical diamorphine use in the UK (Beckett 2007) or cocaine abuse (Beckett 2012). The mistakes we found in his books also fall into the categories widely presented incorrectly. In his novel “Die Verlorenen” (“The Lost”) (Beckett 2022), children get diazepam to numb them for a short time. As diazepam is a long-acting benzodiazepine, this is questionable. Additionally, the use of benzodiazepines in children has always the risk of paradoxical reactions which is not addressed in the book.

However, from a pharmacological point of view, Becketts books go in a direction where the reader can learn something about substances and pharmacological principles without being miseducated. The second British author in the analysis (A.C. Doyle) also performed mostly correct leading to the conclusion that British authors were the most accurate in this analysis.

The Scandinavians J. Nesbø and J.A. Olsen provided the most examples for this analysis which by mere number increases the chance of finding inaccuracies by a closer look. However, the trend is clear that in those books, the focus was not always on educating the viewer and giving most correct presentation.

Overall, the main misconception in the modern books relate to widely known and widely used substances. Most of the readers will get in contact with those substances sooner or later.

It is important to draw a realistic picture of those to increase the acceptance for pharmaceuticals in the general population and give them the best possible treatment.

### Comparison to TV series

There has been research done before regarding pharmacological content in fiction. Two current papers discuss the pharmacological content in the most watched German crime TV-Series “Tatort.” Whereas Ellerbeck and Seifert (2022) mainly focused on quantitative aspects, Borchert and Seifert (2023) concentrated on a reality check. Both is a good basis for comparison with our analysis of the novels as it includes quantitative and qualitative aspects.

The “Tatort” seems to have a greater value for the consumer regarding pharmacology than the novels. Although the mechanism of action was also lacking nearly as often as in our analysis, “Tatort” provides additional information more regularly. When it comes to the correctness of this information the TV series also yielded better results. The analysis taken for the TV was slightly different as it also included professional pharmaceutical use to treat symptoms and heal people.

However, it seems that more careful research was done in preparation of the TV series than for most of the novels. This breaks with the cliché that books are always more informative and better for education than TV series. Authors might benefit from this, finding out on how research is performed for TV and adopt their own processes, so that more correct information reaches their audience. More suggestions for improvement and an overall assessment of the analyzed parameters can be found in Table 4.

**Table 4:** Overall findings and suggestions for future crime novels. This table gives an overview of our overall findings with an assessment and suggestions for improvement

Parameter	Quality of implementation in the crime novels	Reason for quality assessment, suggestions for future novels
Substance presentation	Good	A great variety of substances is presented, especially in the newer novels
Substance application	Good	Common ways of application for the specific time period are shown, the increasing importance of injectable drugs is shown in the newer novels
Outcome of poisoning	Mediocre	The increasing role of criminal anesthesia gets represented in the newer novels but death as major outcome overvalues the danger lies within drugs
Etiology of poisoning cases	Poor	Poisoning by accident is rarely shown although it is the most common way in real life. Authors should raise public awareness for that by presenting this more often
Gender distribution in poisoning cases	Poor	Overall, the gender distribution is not realistically shown. With having a look on data from public services like poison information centers, authors could improve on that
Additional drug information provided	Mediocre	There are some great examples of detailed drug descriptions from every author. If the authors would implement that regularly, readers could learn a lot more about certain substances
Drug mechanism of action provided	Poor	Nearly all books miss the chance to present a mechanism of action. Authors should use this possibility to educate readers on a casual basis
Pharmacological correctness	Mediocre	Although the majority of cases is correct it has decreased over time. Modern authors should do more careful research or use professional pharmacological supervision

## Limitations and conclusions

Of course, we could not read all existing crime literature. However, as our findings for the older novels coincide with the results of the Agatha Christie analysis by Gerald, it can reasonably be concluded that they are representative. Gerald as well described a substantial use of arsenic and strychnine in her stories (Gerald 1993b). The metal arsenic even appeared in one quarter of those (Gerald 1993c). Same coincidence with our findings could be found for way of application, with a huge number of cases including oral application rather than injectable ones (Gerald 1993d). Qualitatively, Gerald outlined the detailed descriptions of drug use in her stories (Gerald 1993e). This again matches our results showing that more additional information on drug use was described in the older novels.

In our selection, the variety of pharmacological content in crime novels is overall large. The reader gets in contact with multiple substances in many ways of application and presentation.

The variety has increased over time showing that some authors like to reflect trends and progress pharmacology has made over the last decades. This especially comes true regarding drugs and hypnotics. Their rise in reality over the past decades is also shown by constant mention in the fictional world. Unfortunately, hypnotics and anesthetics are also the substances which got misrepresented most commonly. The same misconceptions appear repeatedly.

When it comes to etiology and gender, the books did not draw a realistic picture. Accidental poisoning and suicide are massively underrepresented. This might switch readers focus from the serious threat of domestic poisoning. Especially older novels also suggested a safer usage of substances by women which does not correspond to reality.

Regarding the education of the viewers, there is lots of space for improvement. The mechanism of action is rarely presented, and additional information is also missing quite regularly. The lack of plausible description is potentially dangerous as readers might get false ideas of substances and their action principle in general. Although authors of fictious books have artistic freedom, they should consider the impact they have when presenting drugs to the general public.

Great examples provided in some novels and the much higher rate of correct cases in TV series show that this is possible. This could be the first step in the right direction to get better quality novels which educate the readers beyond pure entertainment.

## Supplementary Information

Below is the link to the electronic supplementary material.

Supplementary file1 (DOCX 1721 KB)

## Data Availability

All source data for this work are available upon reasonable request.

## Abbreviations

ADRs: Adverse drug reactions
COVID19: Coronavirus disease 2019
EMCDDA: European Monitoring Centre for Drugs and Drug Addiction
GABA: Gamma-aminobutyric acid
GIZ: Giftinformationszentrum (“poison information center“)
UNDOC: United Nations Office on Drugs and Crime
