# Supplementary material for: Presentation of pharmacological content in crime novels between 1890 and 2023
Source: Naunyn Schmiedebergs Arch Pharmacol. 2024 Apr 21;397(9):7089–102. doi: 10.1007/s00210-024-03103-w (PMC11422460; doi:10.1007/s00210-024-03103-w)
Supplement: Supplementary file 1 — Supplementary file1 (DOCX 1721 KB) [file 210_2024_3103_MOESM1_ESM.docx]

**Supplemental Figures S 1 - S 11 and Table S1**

**Iven H. Moeller and Roland Seifert**

**Presentation of pharmalogical content in crime novels between 1890 and 2023**

| Book author – English title/title of the German translation | Number of pages |
| --- | --- |
| J. Nesbø – The Bat/Der Fledermausmann | 432 |
| J. Nesbø – Cockroaches/Kakerlaken | 432 |
| J. Nesbø – The/Redbreast/Rotkehlchen | 480 |
| J. Nesbø – Nemesis/Die Fährte | 576 |
| J. Nesbø –  The Devil's Star/Das fünfte Zeichen | 496 |
| J. Nesbø – The Redeemer/Der Erlöser | 528 |
| J. Nesbø – The Snowman/Schneemann | 512 |
| J. Nesbø – The Leopard/Leopard | 704 |
| J. Nesbø – Phantom/Die Larve | 576 |
| J. Nesbø – Police /Koma | 624 |
| J. Nesbø – The Thirst/Durst | 624 |
| J. Nesbø – Knife/Messer | 576 |
| J. Nesbø – Killing Moon/Blutmond | 544 |
| J. Nesbø – Blood on Snow/Blood on Snow der Auftrag | 192 |
| J. Nesbø – Blood on Snow/Blood on Snow das Versteck | 256 |
| J. Nesbø – Headhunters/Headhunter | 320 |
| J. Nesbø – The Son/Der Sohn | 528 |
| J. Nesbø – MacBeth/MacBeth | 624 |
| J. Nesbø – The Jealousy Man/Eifersucht | 272 |
| J. Nesbø – The Kingdom/Ihr Königreich | 592 |
| S. Beckett – The Chemistry of Death/Die Chemie des Todes | 432 |
| S. Beckett – Written in bone/Kalte Asche | 432 |
| S. Beckett – Whispers of the Dead/Leichenblässe | 416 |
| S. Beckett – The Calling of the Grave /Verwesung | 443 |
| S. Beckett – Cat and Mouse/Katz und Maus | 64 |
| S. Beckett – The Restless Dead/Totenfang | 560 |
| S. Beckett – The Scent of Death/Die ewigen Toten | 480 |
| S. Beckett – The Lost /Die Verlorenen | 416 |
| S. Beckett – Animals/Tiere | 288 |
| S. Beckett – Where There's Smoke/Flammenbrut | 400 |
| S. Beckett – Voyeur/Voyeur | 380 |
| S. Beckett – Stone Bruises/Der Hof | 464 |
| S. Beckett – Obsession/Obsession | 416 |
| S. Beckett – Snowfall & Just Another Day /Schneefall & Ein ganz normaler Tag | 64 |
| S. Beckett – not available/Versteckt: dunkle Geschichten | 144 |
| J.A. Olsen – Mercy/Erbarmen | 432 |
| J.A. Olsen – Disgrace /Schändung | 464 |
| J.A. Olsen – Redemption/Erlösung | 592 |
| J.A. Olsen – Journal 64/Verachtung | 560 |
| J.A. Olsen – Buried/Erwartung | 576 |
| J.A. Olsen – The Hanging Girl/Verheißung | 608 |
| J.A. Olsen – The Scarred Women/Selfies | 576 |
| J.A. Olsen – Victim 2117/Opfer 2117 | 592 |
| J.A. Olsen – The Shadow Murders /Natrium Chlorid | 528 |
| J.A. Olsen – The Alphabet House/Das Alphabethaus | 592 |
| J.A. Olsen – The Washington Decree/Washington Dekret | 648 |
| J.A. Olsen – Takeover/Takeover | 608 |
| J.A. Olsen – not available/Miese kleine Morde | 138 |
| Sir A.C. Doyle – The Adventure of the Devil's Foot/Der Teufelsfuß | 32 |
| Sir A.C. Doyle – The Sign of the Four /Das Zeichen der Vier | 143 |
| Sir A.C. Doyle – The adventures of Sherlock Holmes/Die Abenteuer des Sherlock Holmes | 386 |
| G. Simenon – Maigret and the Old Lady/Maigret und die alte Dame | 196 |
| G. Simenon – The Iron Stairs /Die Eisentreppe | 197 |
| G. Simenon – Maigret has Scruples/Maigret hat Skrupel | 208 |
| G. Simenon – The Man in the Street/Maigret und der Mann auf der Straße | 102 |
| G. Simenon – Mit Maigret in die Bretagne | 390 |
| G. Simenon – The 13 Culprits/13 Man lernt nie aus | 125 |
| G. Simenon – The Truth About Bebe Dong/Die Warheit über Bebe Dong | 182 |
| G. Simenon – Sunday/Sonntag | 148 |
| G. Simenon – Emile's Boat/Emil und sein Schiff | 255 |
| G. Simenon – unavailable/Aus den Akten der Agence O | 330 |

**Table S1:** The table lists all books that were read for this research. It includes author, German title and the number of pages.

**
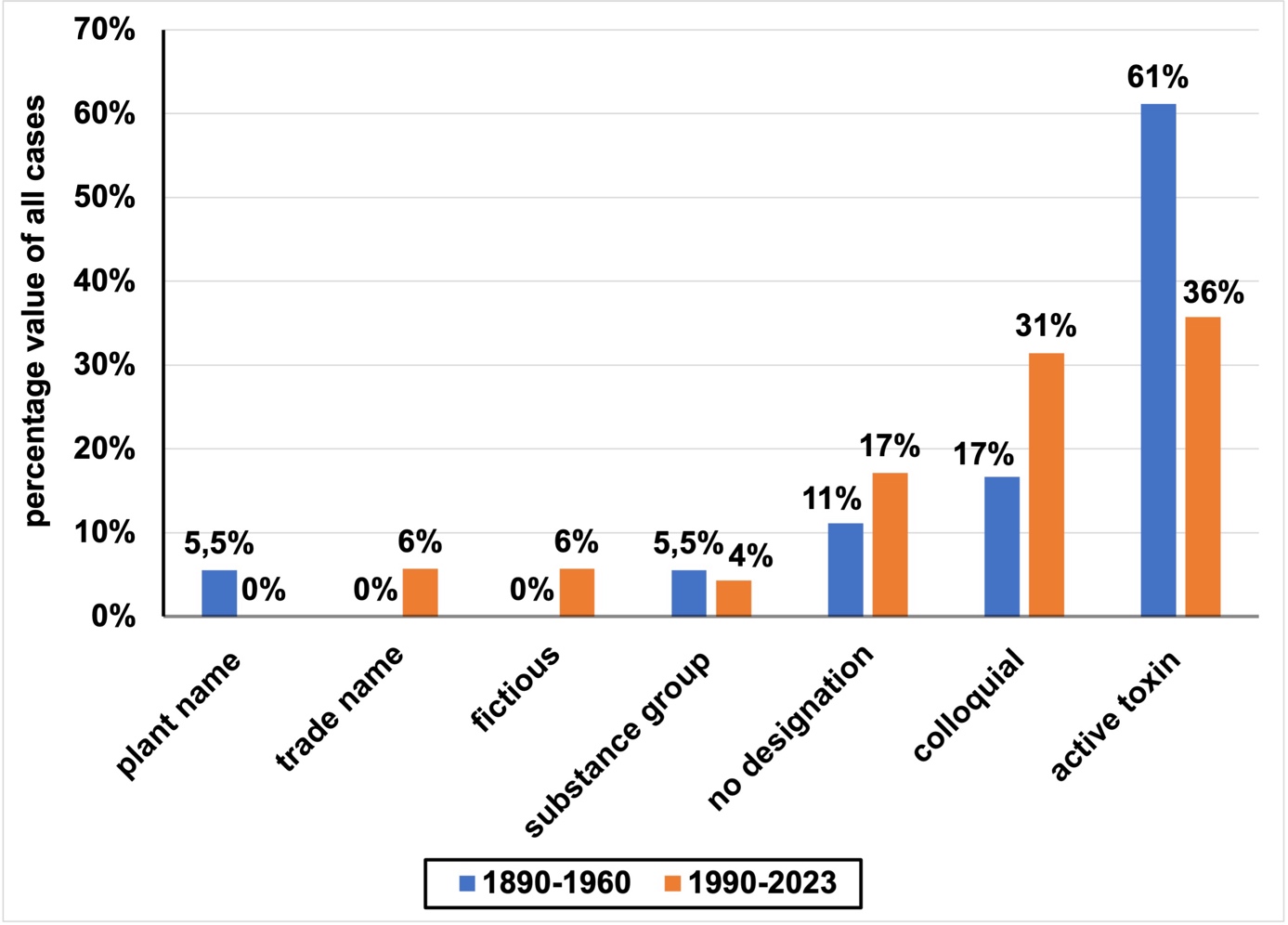
**

**Figure S1:** Designation of the active substances in older and newer crime literature shown in a bar chart.


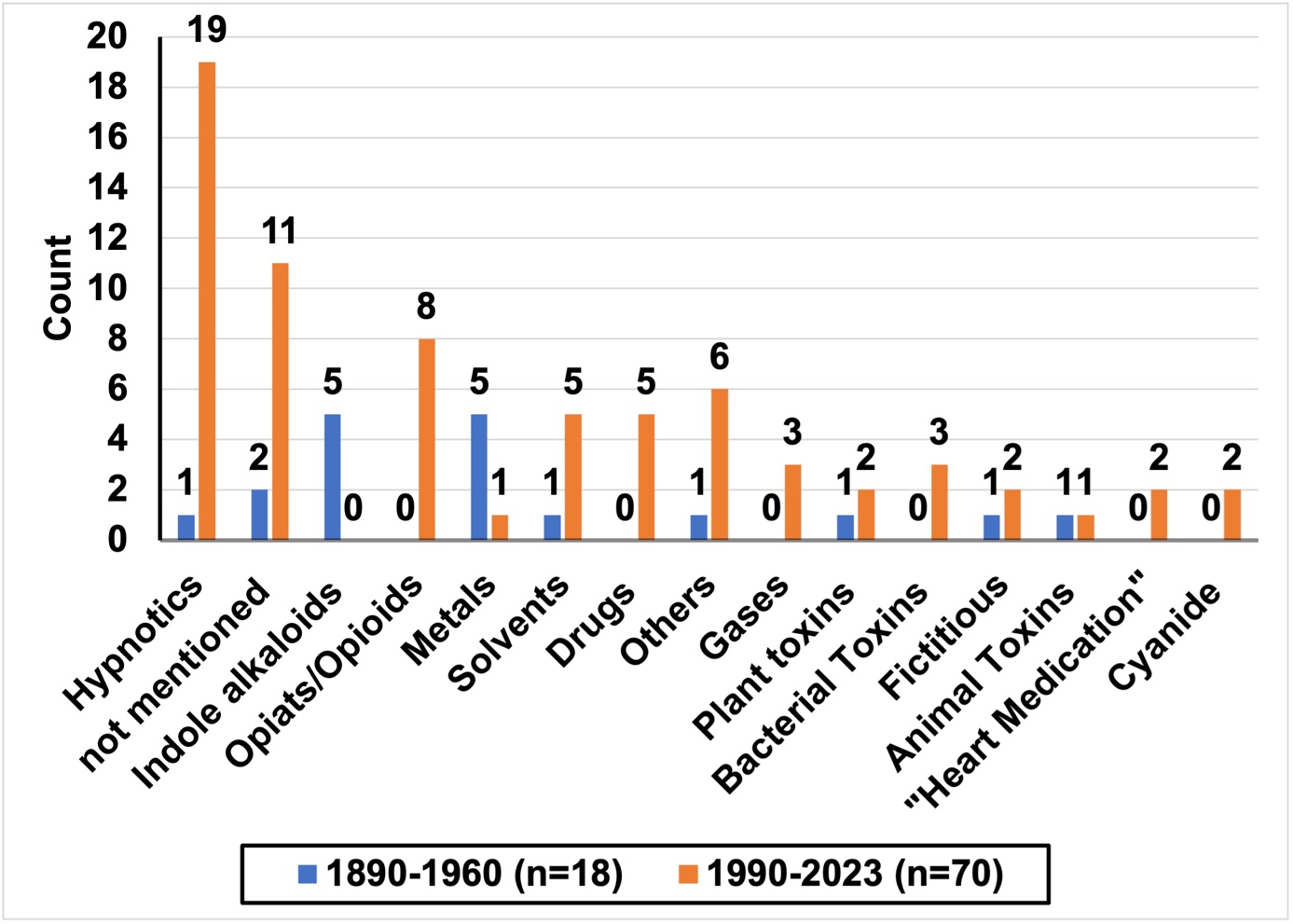


**Figure S2:** Substance Categories by absolute count divided into older and newer books. The values for the newer novels are shown by the orange columns. The values for the older novels are shown by the blue columns.


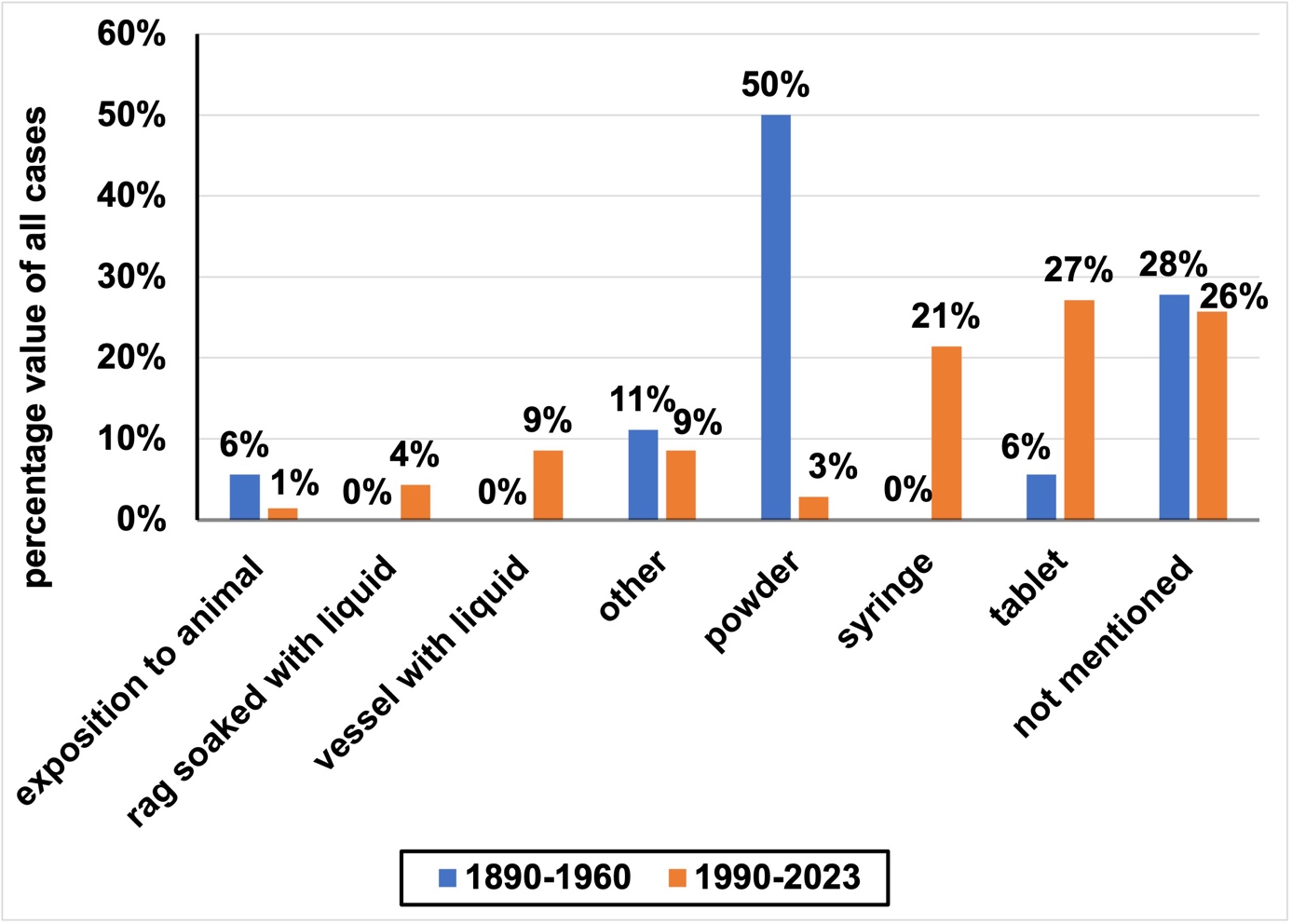


**Figure S3:** Substance presentation in older and newer crime literature shown in bar chart.


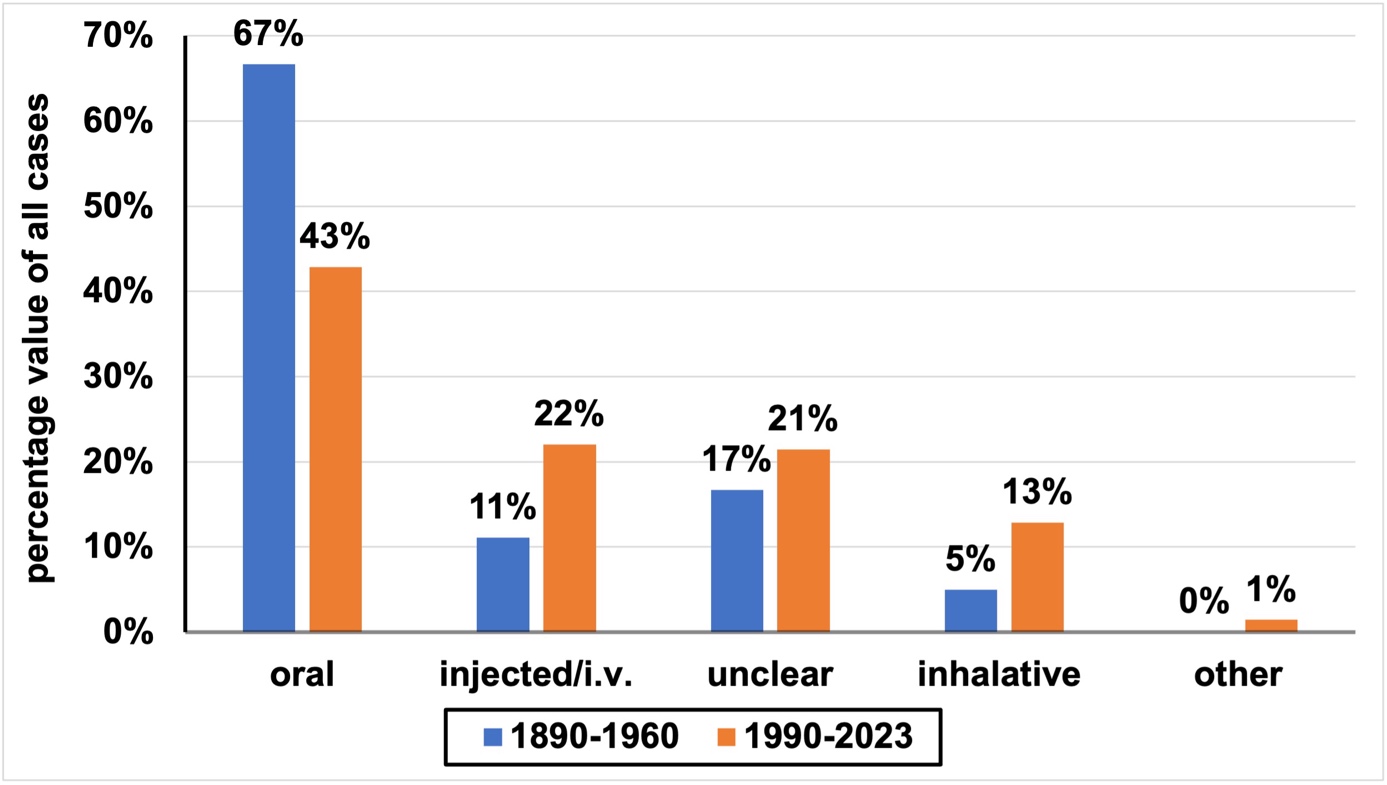


**Figure S4:** Application of poisons in older and newer literature, bar chart.


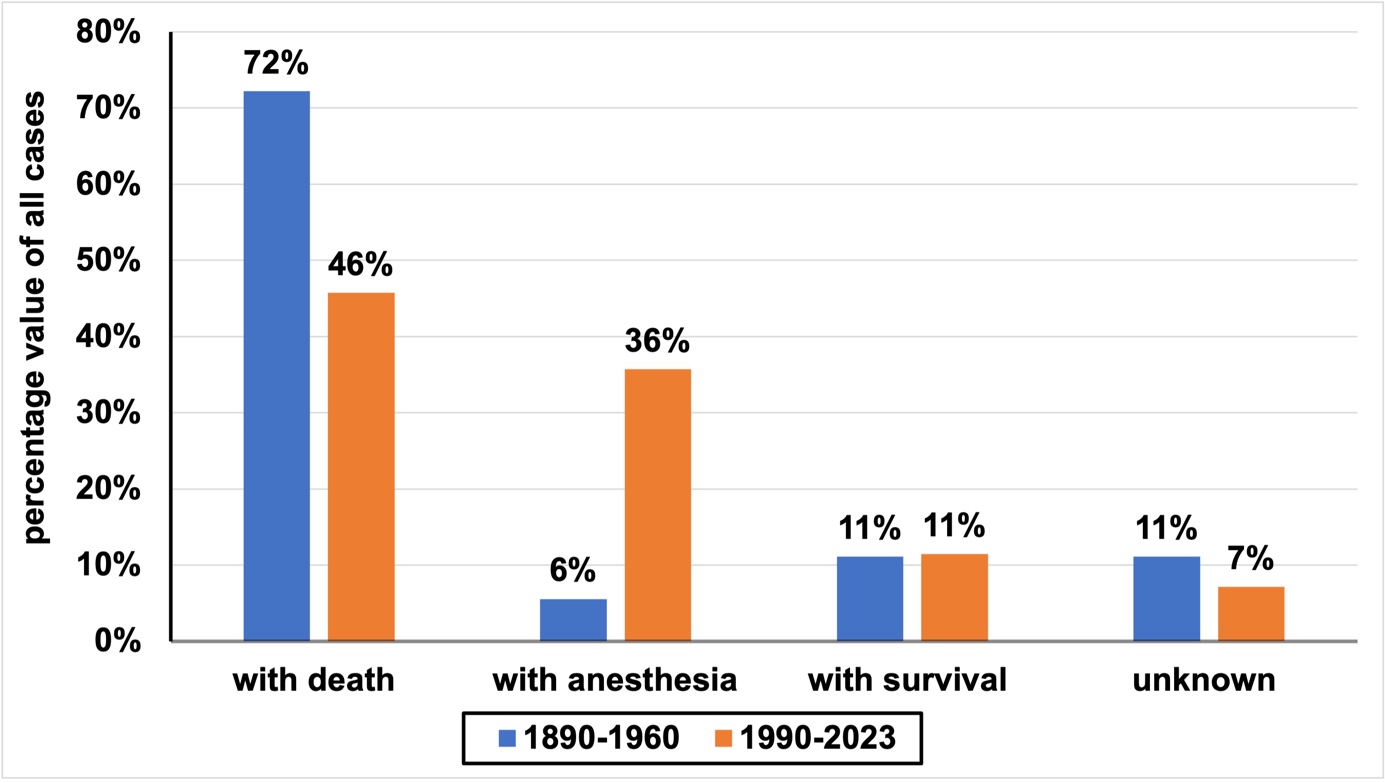


**Figure S5:** Outcome of Poisoning in all analyzed cases shown in a bar chart.


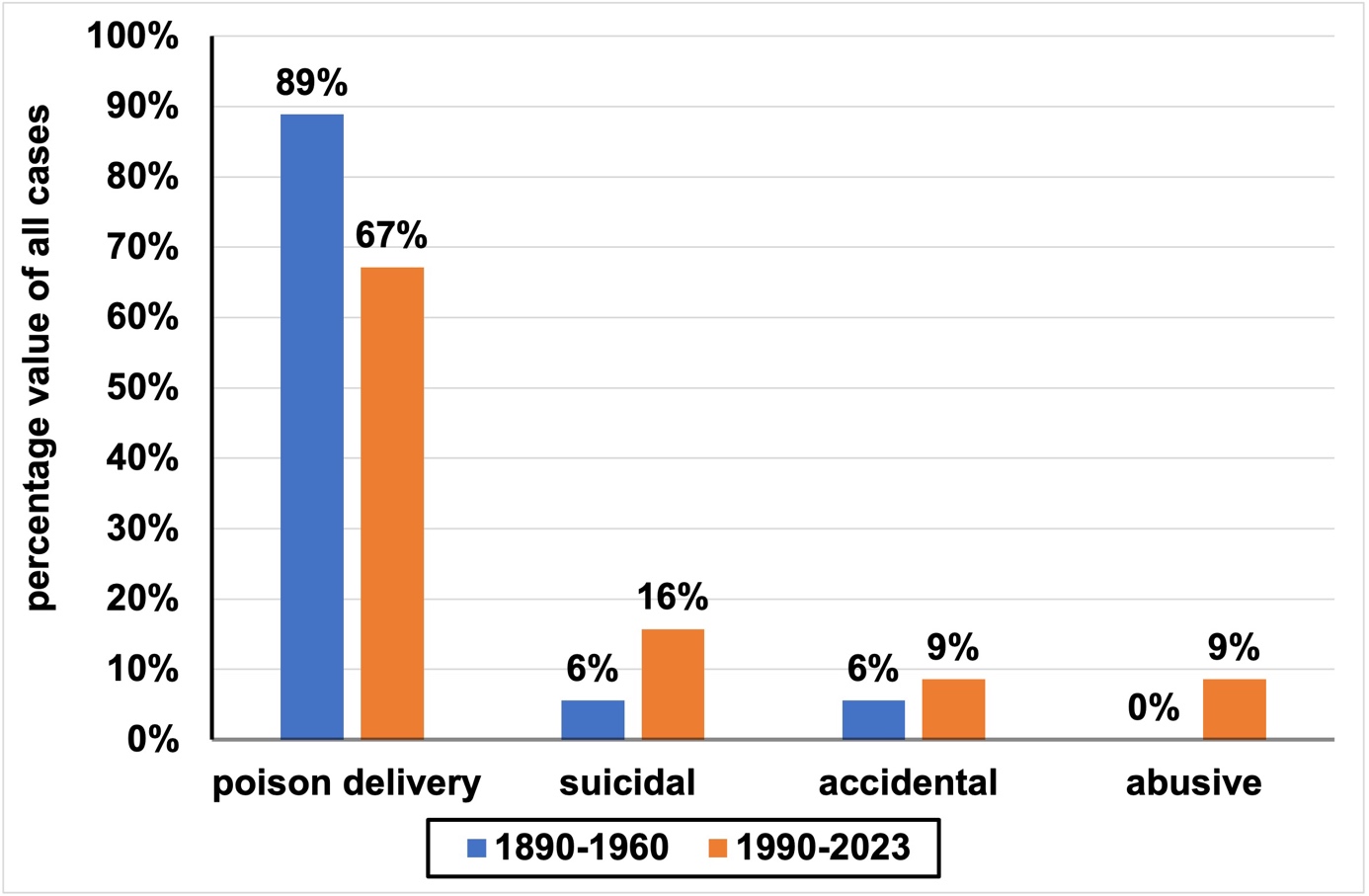


**Figure S6:** Etiology of poisoning in comparison of older and newer cases, shown in a bar chart.


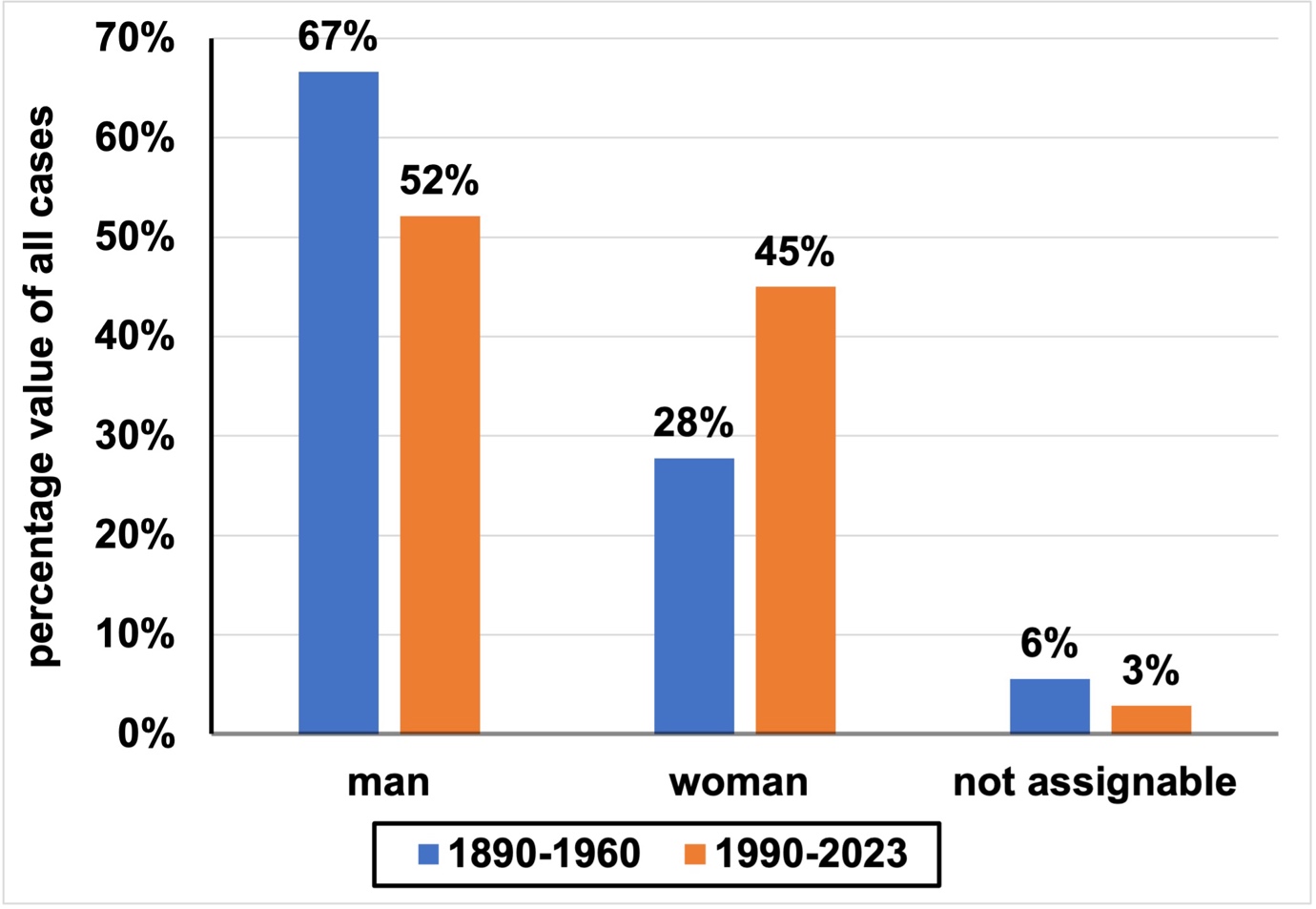


**Figure S7:** Gender distribution for victims of fictional poisoning in comparison of older and newer novels and reality in a bar chart.

**
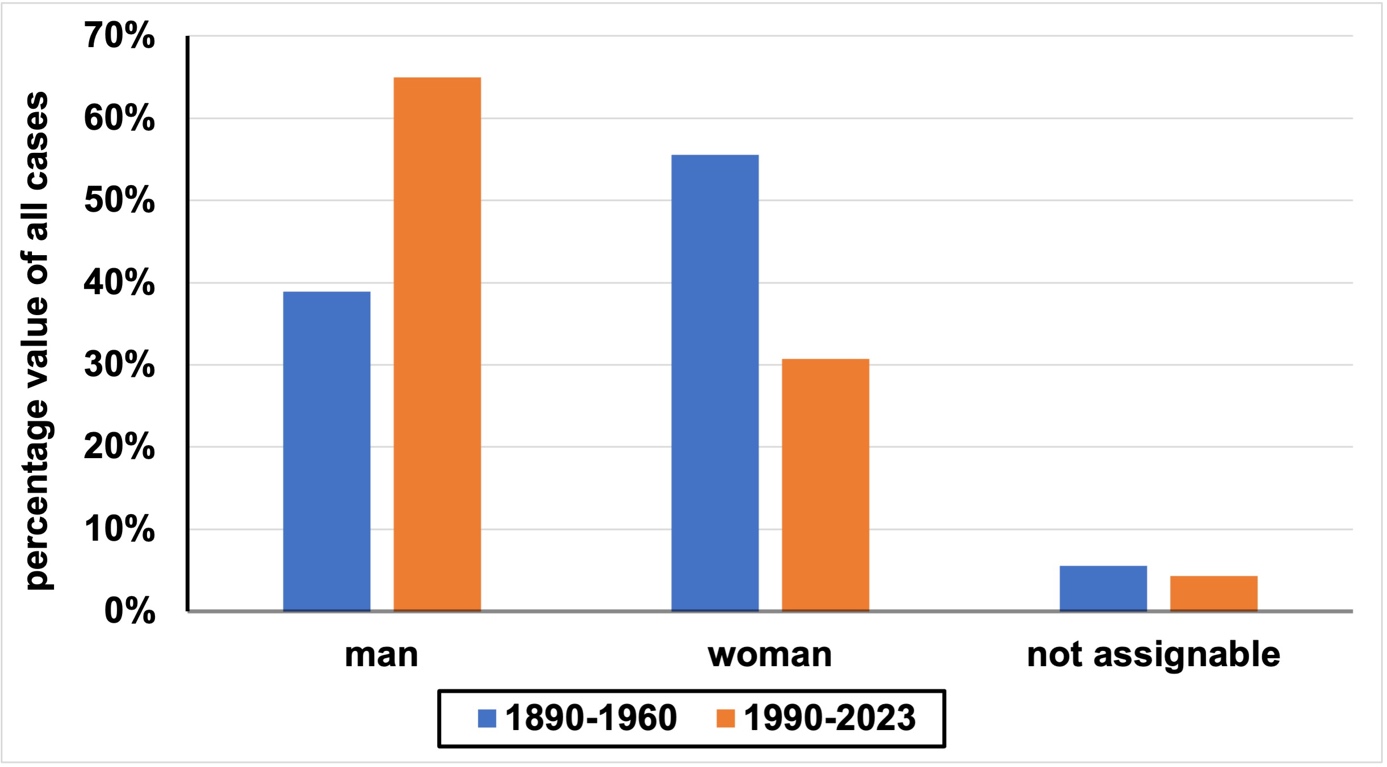
**

**Figure S8:** Gender distribution for offenders of fictional poisoning in comparison of older and newer novels and reality in a bar chart.


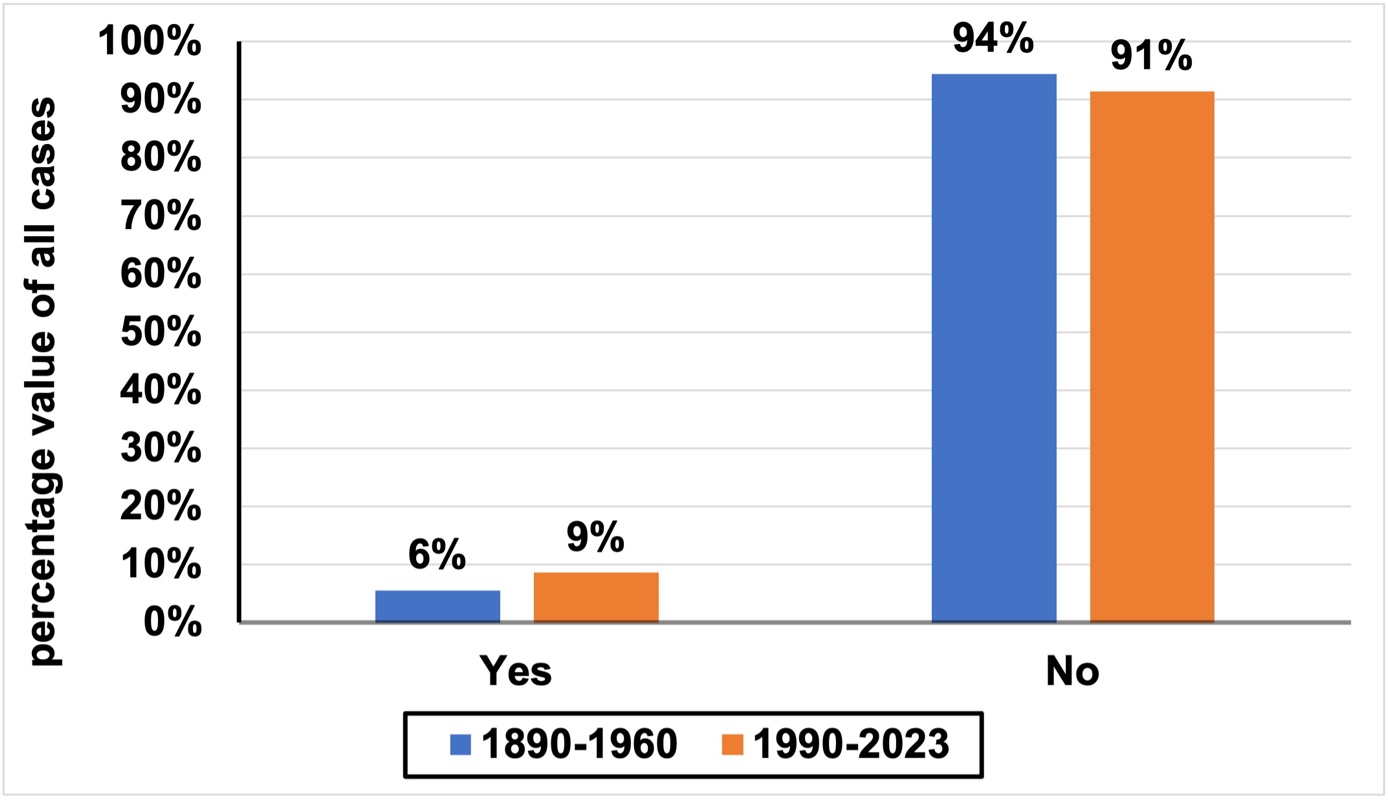


**Figure S9:** Mechanism of action provided – The bar chart compares older and newer novels regarding the question if a mechanism of action is provided or not.


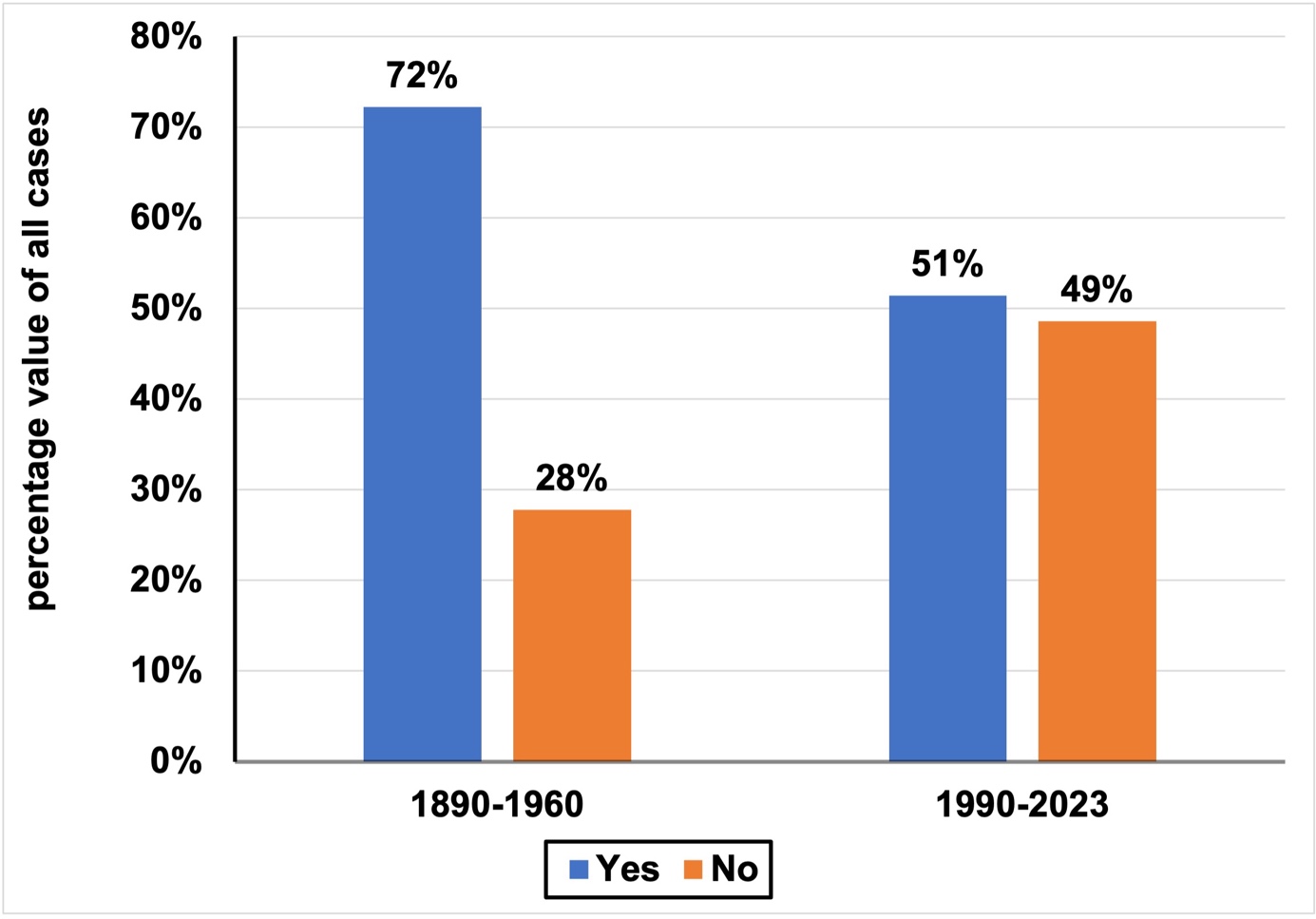


**Figure S10:** Additional Information provided. The bar chart shows the percentage of all analyzed novels provided additional information regarding the use pharmacological substances and those that did not.


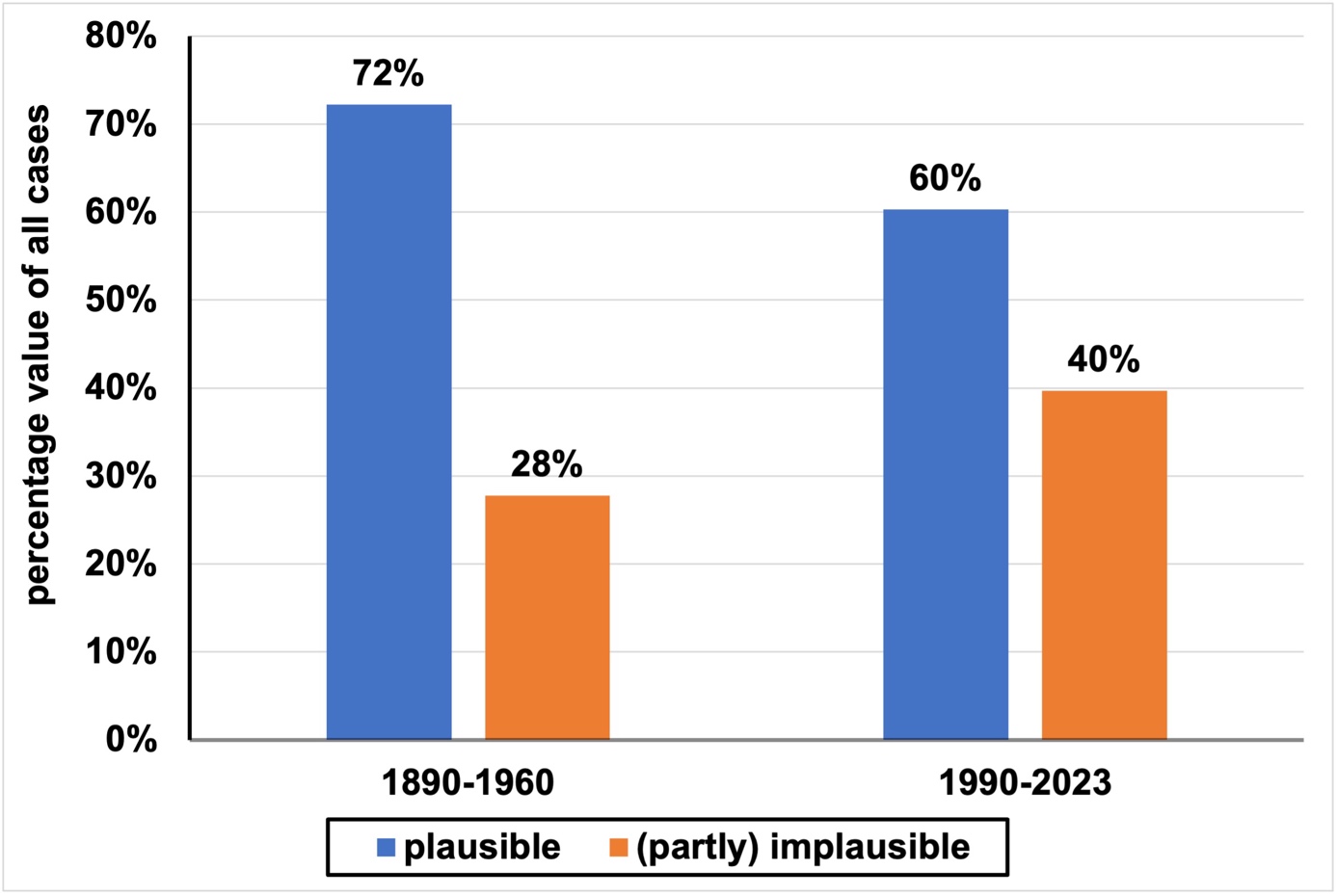


**Figure S11:** The bar chart shows the pharmacological plausibility for the cases found in the old novels on the left-hand side and those for the newer ones on the right-hand side.
